# Chinese medicine-based interventions in triple-negative breast cancer: mechanistic evidence, clinical findings, and translational challenges-a narrative review

**DOI:** 10.3389/fphar.2026.1787613

**Published:** 2026-09-09

**Authors:** Guoli Feng, Changju Chen, Taolang Li

**Affiliations:** Affiliated Hospital of Zunyi Medical University, Zunyi, China

**Keywords:** Chinese medicine-based interventions, clinical trials, evidence appraisal, ferroptosis, model heterogeneity, translational research, triple-negative breast cancer

## Abstract

Triple-negative breast cancer (TNBC) is an aggressive and heterogeneous breast cancer subtype with early recurrence and limited therapeutic targets. The evidence reviewed here spans several non-equivalent intervention classes: licensed or commercial multi-component Chinese medicine products, standardized trial formulations, experimental botanical mixtures, and isolated constituents, fractions, or derivatives. We use the umbrella term Chinese medicine-based interventions (CMBIs) only for evidence mapping and do not assume pharmacological, manufacturing, or regulatory equivalence among these classes. Evidence was appraised on two independent dimensions: relevance to TNBC and study design/methodological robustness. TNBC-specific clinical evidence is limited to a small number of randomized and observational studies with dissimilar endpoints; a 2022 meta-analysis reported favorable pooled estimates but also substantial methodological weaknesses and intervention heterogeneity. Preclinical findings suggest pathway-associated effects involving apoptosis, ferroptosis, immune regulation, epithelial-mesenchymal transition, metabolism, and chemotherapy response. Ferroptosis evidence is currently limited to preclinical Shuganning injection studies showing iron-dependent lipid peroxidation and ferrostatin-1-sensitive cell death, without clinical validation in TNBC. Overall, CMBIs should be regarded as investigational adjunctive approaches rather than established TNBC-specific therapies. Priorities include product-level chemical characterization, clinically relevant exposure assessment, model-diverse causal validation, multicenter trials, and preparation-specific safety and interaction monitoring.

## 1 Introduction

Triple-negative breast cancer (TNBC) is a biologically distinct and highly aggressive subtype of breast cancer characterized by the absence of estrogen receptor (ER), progesterone receptor (PR), and human epidermal growth factor receptor 2 (HER2) expression. Accounting for approximately 15%–20% of all breast cancer cases, TNBC is associated with a poorer prognosis compared to other breast cancer subtypes due to its high invasiveness, early recurrence, and limited targetable options (Guadagni et al., 2024; Almansour, 2022). Although the treatment landscape of TNBC has expanded with chemotherapy, immunotherapy, PARP inhibitors, and antibody-drug conjugates, chemotherapy remains an important backbone of systemic treatment in many clinical settings (Bianchini et al., 2022; Wen et al., 2024). However, treatment resistance and therapy-related toxicity continue to complicate disease management. Consequently, there remains a clinical need to explore adjunctive and complementary strategies that may improve treatment tolerance, symptom control, and therapeutic outcomes.

From a molecular perspective, TNBC exhibits considerable heterogeneity and complex pathogenesis involving multiple signaling pathways and genetic alterations. Key molecular events implicated in TNBC progression include dysregulation of epithelial-mesenchymal transition (EMT), activation of oncogenes and tumor suppressor genes, aberrant epigenetic modifications, and remodeling of the tumor microenvironment (Tong et al., 2022; Chen et al., 2022; Wu et al., 2024). For example, TMEM158 has been shown to promote EMT and metastasis via TGF-β pathway activation in TNBC cells (Tong et al., 2022), while ICAM1 facilitates bone metastasis through integrin-mediated TGF-β/EMT signaling (Chen et al., 2022). Moreover, epigenetic regulators such as histone deacetylase 6 (HDAC6) play a critical role in TNBC tumorigenesis, making them promising therapeutic targets (Guadagni et al., 2024). The complex interplay of these molecular alterations underlies the aggressive phenotype and therapeutic resistance characteristic of TNBC (Almansour, 2022).

Traditional Chinese medicine is used in China as an adjunctive approach in cancer care, but the interventions discussed in this literature are not a single pharmacological or regulatory category. For clarity, this review uses Chinese medicine-based interventions (CMBIs) as a descriptive umbrella comprising four distinct classes: (1) licensed or commercial multi-component products, such as Compound Kushen injection and Shuganning injection; (2) standardized trial formulations, such as Sanyin Formula; (3) experimental multi-botanical mixtures; and (4) isolated constituents, enriched fractions, or derivatives, such as matrine, oxymatrine, cinobufagin, cardamonin, Huaier polysaccharides, and sophoridine derivatives. An isolated compound or fraction is not treated as evidence for a complete formulation, and an experimental formula is not assumed to represent an approved commercial product.

The Chinese medicine literature relevant to TNBC is scattered across these intervention classes and across clinical, animal, cell, and computational studies (Wu et al., 2024; Zhao et al., 2021; Yang et al., 2021). Combining these sources without explicit labeling can obscure differences in product identity, exposure, model validity, and evidentiary strength. This review therefore distinguishes intervention class, product status, TNBC relevance, and study design throughout the synthesis; formulation-level conclusions are limited to studies that evaluated the complete preparation.

This narrative review critically summarizes mechanistic evidence, clinical findings, safety data, and translational barriers for CMBIs in TNBC. The review does not treat TNBC relevance as an evidence hierarchy. Instead, each study is considered along two independent dimensions: (1) relevance to TNBC and (2) design and methodological robustness. Mechanistic claims are described as pathway-associated unless supported by direct target engagement, genetic perturbation or rescue, selective pharmacology, or another causal validation strategy.

**FIGURE 1:**
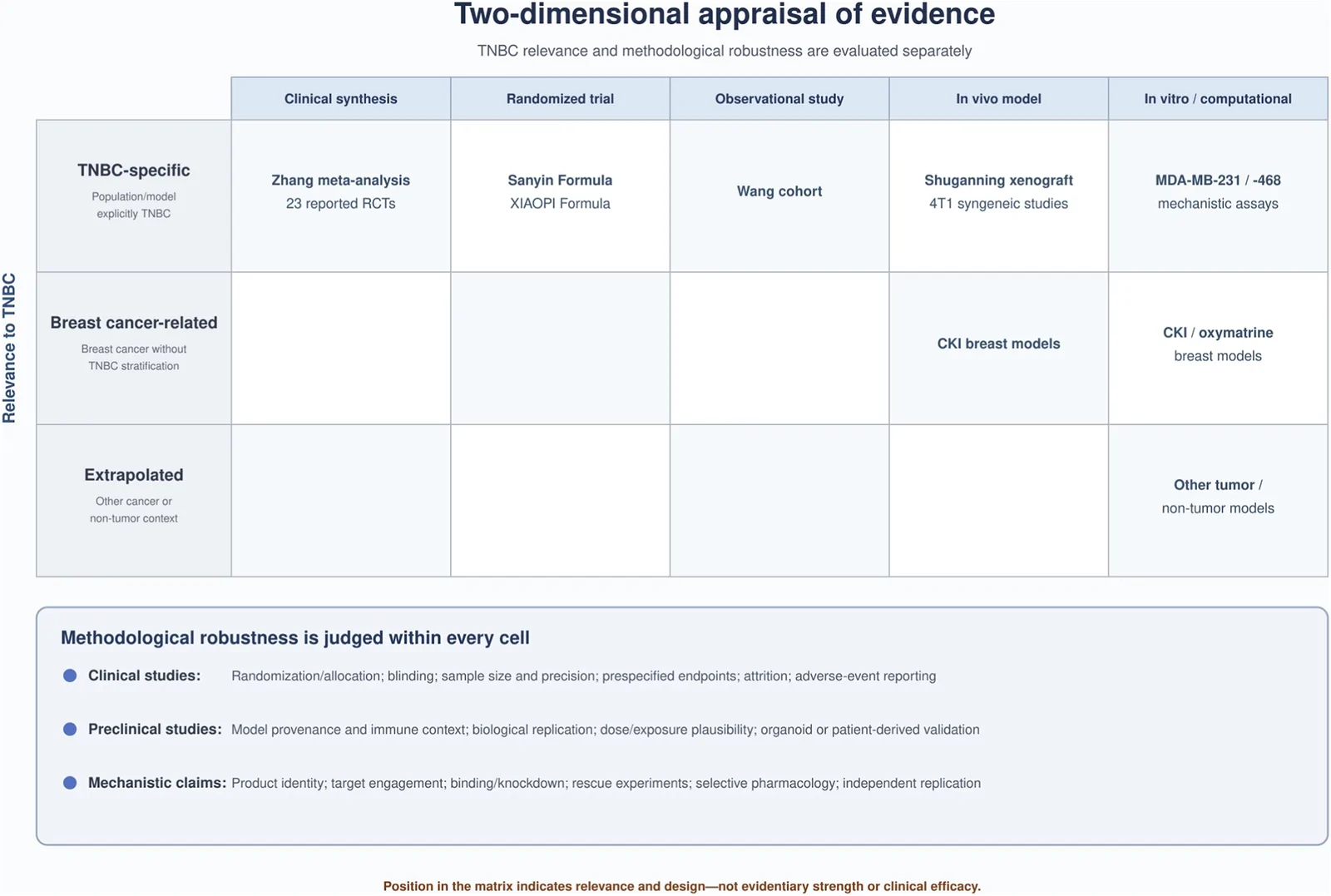
Two-dimensional appraisal of evidence for Chinese medicine-based interventions in TNBC. Rows classify relevance to TNBC (TNBC-specific, breast cancer-related, or extrapolated), whereas columns classify study design (clinical synthesis, randomized trial, observational study, *in vivo* model, and *in vitro*/computational evidence). Methodological robustness is judged within each cell using design-appropriate criteria. Matrix position indicates relevance and design, not evidentiary strength or clinical efficacy. CKI, Compound Kushen injection; RCT, randomized controlled trial; TNBC, triple-negative breast cancer.

Figure 1 presents this two-dimensional framework. Rows indicate TNBC-specific, breast cancer-related, or extrapolated relevance; columns identify study design. Methodological robustness is then judged within each cell using design-appropriate criteria. Thus, a TNBC-specific cell experiment is highly relevant to the disease context but remains lower in clinical inferential strength than a well-conducted randomized trial.

## 2 Methods

This narrative review integrated heterogeneous evidence on licensed/commercial multi-component Chinese medicine products, standardized trial formulations, experimental botanical mixtures, and isolated constituents, fractions, or derivatives in TNBC. These classes were not considered interchangeable. Narrative synthesis was selected because interventions, experimental systems, and outcome measures were too heterogeneous for a single quantitative analysis (Sukhera, 2022; Baethge et al., 2019).

Literature was searched in PubMed, Web of Science, Embase, Google Scholar, CNKI, Wanfang, and SinoMed from database inception to 30 May 2026. Search terms combined “triple-negative breast cancer” or “TNBC” with “Chinese medicine”, “Chinese medicine formula”, “Chinese medicine preparation”, the names of complete products or formulas (including Compound Kushen injection, Sanyin Formula, XIAOPI Formula, and Shuganning injection), isolated constituents or fractions, and mechanism terms including “ferroptosis”, “apoptosis”, “immune microenvironment”, “epithelial-mesenchymal transition”, “PI3K/AKT”, “STAT3”, “angiogenesis”, “metabolic reprogramming”, and “chemotherapy resistance.” Reference lists of key reviews and original studies were also screened.

Studies were considered relevant if they addressed a defined Chinese medicine-based intervention in TNBC or breast cancer. The intervention was first classified as a licensed/commercial multi-component product, standardized trial formulation, experimental botanical mixture, or isolated constituent/fraction/derivative. Evidence relevance was then classified as TNBC-specific, breast cancer-related without explicit TNBC stratification, or extrapolated from another tumor or non-tumor context. Extrapolated evidence was used only for mechanistic plausibility and not as direct evidence of TNBC efficacy.

A separate design and methodological appraisal considered whether evidence arose from a systematic review/meta-analysis, randomized trial, observational study, *in vivo* model, *in vitro* model, or computational analysis. Clinical appraisal considered randomization, allocation concealment, blinding, sample size and precision, attrition, endpoint prespecification, confounding, and adverse-event reporting. Preclinical appraisal considered human versus murine origin, immune-competent versus immune-deficient context, primary versus metastatic derivation, molecular phenotype, two-dimensional culture versus organoid or patient-derived models, dose/exposure plausibility, product identity, causal validation, and independent replication. Because this was a narrative review, RoB 2, ROBINS-I, SYRCLE, and formal certainty grading were not applied; the resulting judgments are structured narrative appraisals rather than formal scores.

For botanical drugs or plant-derived metabolites discussed in the manuscript, taxonomic names were checked against recognized botanical nomenclature sources where applicable. Full species names, authorities, families, and relevant preparation or metabolite information are provided in Supplementary Table 1. The reporting and ethnopharmacological transparency of this narrative review were assessed using the relevant sections of the ConPhyMP/GA Best Practice guidance (Heinrich et al., 2022). The completed checklists are provided as separate supporting files named GA-BP Table 1 Table 2. Supplementary Table S1, S2.

**TABLE 1:** Product-level and two-dimensional appraisal of principal Chinese medicine-based interventions discussed in TNBC.

Intervention	Intervention class/status	Product-level identity	TNBC relevance/model	Design and methodological robustness	Reported pathway-associated finding	Causal validation/major limitation	Key refs
Compound Kushen injection	Commercial multi-component injectable product	Complete preparation from Radix Sophorae Flavescentis (Kushen) and Rhizoma Heterosmilacis (Baituling); multiple alkaloids including matrine/oxymatrine	Breast cancer-related; some MDA-MB-231 experiments	Cell, multi-omics, and computational studies; no direct TNBC clinical trial in reviewed set	Cell-cycle, apoptosis, metabolism, DNA repair, tumor environment	Many targets computational/pathway-associated; isolated alkaloid data not equivalent; batch/exposure details incomplete	Cui et al. (2020); Li et al. (2021); Cui et al. (2019); (Liu et al. 2022)
Shuganning injection	Commercial multi-component injectable product; licensed for hepatic indications, not TNBC	Complete standardized injectable patent medicine; oncology use investigational	TNBC cells and nude-mouse xenograft	Preclinical *in vitro*/*in vivo* program; immune-deficient host; no TNBC clinical evidence	HO-1-associated Fe2+ accumulation, ROS/lipid peroxidation, ferroptotic death	Ferrostatin-1 and HO-1 dependence strengthen mechanism; no direct target binding or human validation	Du et al. (2021)
Sanyin Formula	Standardized trial formulation; not assumed to be a marketed oncology product	Nine-herb granules; manufacturer, 14 g twice-daily dose, and ≥2-year duration reported	Direct TNBC clinical RCT; TNBC metastasis mouse study	Multicenter double-blind RCT plus preclinical study; modest clinical sample and incomplete subtype detail	DFS signal; JAK/STAT3-associated paclitaxel response in mice	Clinical efficacy requires replication; mouse pathway change does not establish a direct target	Wang et al. (2023); Wu et al. (2022)
XIAOPI Formula	Multi-component product/formulation; Chinese approval for mammary hyperplasia, not TNBC	Ten-material reflux extract; HPLC fingerprint reported; 8.5 g three times daily in trial	Direct TNBC clinical trial plus MDA-MB-231/4T1/macrophage models	Small randomized trial; mixed human/murine systems; exploratory DFS	CXCL1-associated immune and stem-cell effects	CXCL1 manipulation supports pathway causality; clinical safety and replication remain limited	Wang et al. (2019); Guo et al. (2024)
Four-botanical formula	Experimental botanical mixture	Laboratory formula combined with doxorubicin; not an approved commercial product	4T1 syngeneic murine model	Immune-competent mouse study; no human validation	Immunoregulatory and antitumor readouts	Murine immune context informative but not a human TNBC subtype; product standardization uncertain	Kan et al. (2024)
Matrine/oxymatrine	Isolated alkaloids; constituents associated with Kushen products	Single compounds, not Compound Kushen injection	MDA-MB-231/-468 or broader breast cancer cells	Predominantly 2D cell studies; limited model diversity	PI3K/AKT-associated apoptosis; αVβ3/FAK/PI3K/AKT-associated EMT	Reduced signaling does not prove direct kinase/integrin inhibition; formulation equivalence invalid	Wei et al. (2023); Chen et al. (2019)
Sophoridine derivatives	Isolated/synthetic derivatives	Derivative series, not a complete formula or commercial preparation	TNBC preclinical models	Cell/animal preclinical evidence; no clinical product	Apoptosis/autophagy with reduced mTOR signaling	Autophagic function and direct target engagement insufficiently resolved	Dai et al. (2022)
Cinobufagin	Isolated bufadienolide associated with Huachansu/Cinobufacini products	Single compound; not equivalent to a complete toad-venom preparation	TNBC preclinical models	Preclinical mechanistic study; no preparation-level clinical confirmation	Macrophage reprogramming; FAK/STAT3-associated effects	Compound-specific; immune model and clinical translation require validation	Zhu et al. (2024)
Huaier polysaccharides	Isolated/enriched fraction	Polysaccharide fraction; not equivalent to a complete commercial Huaier product	TNBC cells/animal models	Preclinical mechanistic evidence	Autophagic degradation of Snail and EMT suppression	Fraction identity/exposure and product equivalence limit translation	Tian et al. (2021)
Cardamonin	Isolated natural compound	Single chalcone; not a polyherbal product	Breast cancer-related models	Preclinical cell/animal evidence; not TNBC-specific clinical evidence	HIF-1α-associated metabolic reprogramming	No product-level or clinical TNBC inference	Jin et al. (2019)

The four intervention classes are non-equivalent. TNBC, relevance is evaluated separately from study design and methodological robustness. Pathway-associated findings are not described as direct target inhibition unless target engagement or causal validation was demonstrated.

**TABLE 2:** Clinical evidence for Chinese medicine-based interventions in TNBC, with separate appraisal of TNBC relevance and methodological robustness.

Study/intervention	TNBC relevance	Design/methodological tier	Population/sample	Intervention/comparator	Follow-up and principal findings	Preparation-specific adverse events	Methodological concerns	Product identity/replicability
Wu et al. (2022) — Sanyin Formula	Direct TNBC clinical evidence	Multicenter randomized, double-blind, placebo-controlled trial	252 women with operable primary invasive TNBC after surgery; 127 vs. 125	Sanyin Formula granules 14 g twice daily plus standard chemotherapy vs. matched placebo plus chemotherapy; ≥2 years	Minimum 42 months; median 51 months. Five-year DFS 94.2% vs. 85.5% (HR 0.40, 95% CI 0.17–0.97; P = 0.034). OS not significantly different	Treatment-related AEs: 3/127 vs. 4/125. Sanyin: upper-limb edema, pain, diarrhea. Placebo: liver-function injury, weakness, pain, diarrhea. No treatment-related deaths or life-threatening events	Relatively strongest direct study, but modest sample; chemotherapy not fully uniform; insufficient OS follow-up; no TNBC molecular-subtype analysis	Nine-herb standardized granules supplied by Tianjiang Pharmaceutical; trial dose and duration reported. Batch-level chemical fingerprint not reported in the article
Guo et al. (2024) — XIAOPI Formula	Direct TNBC clinical and biomarker-linked evidence	Single-center randomized, double-blind, placebo-controlled trial	60 randomized after surgery and chemotherapy; 30 vs. 30; analyzed 28 vs. 30	XIAOPI Formula 8.5 g three times daily vs. matched placebo for 3 months	CXCL1 and patient-reported outcomes improved after 3 months. Exploratory 60-month relapse/metastasis: 5 vs. 6; deaths: 0 vs. 2; DFS P < 0.05	Specific AE counts, categories, and grades not reported; authors stated there were few side effects	Small, single-center study; two losses in treatment arm; subjective outcomes; DFS exploratory and potentially affected by post-trial use; incomplete safety reporting	Ten-material reflux extract with HPLC fingerprinting in the preclinical product report (Wang et al., 2019). Chinese regulatory approval was for mammary hyperplasia, not TNBC.
Wang et al. (2020) — individualized TCM formulas	Direct TNBC observational evidence	Prospective cohort; non-randomized exposure	356 enrolled; 154 followed; 148 analyzed: exposure 73, non-exposure 75	Individualized formulas plus standardized treatment vs. standardized treatment without formula; visits every 3 months	Planned 2-year IDFS; median 18 months. Recurrence/metastasis 8/73 vs. 13/75; adjusted association OR 0.89 (95% CI 0.37–0.956; P < 0.05)	6/73 (8.2%): one severe HILI (RUCAM 6), two mild transaminase elevations, two stomach pain, one diarrhea. No AE-related withdrawal	High concern: non-randomized, confounding/selection bias, substantial attrition, target sample not reached, open label, formula modification reduces reproducibility	Patient-specific formula modification; no single commercial product or uniform batch. Exposure is not replicable as one preparation
Zhang et al. (2022) — formula plus chemotherapy meta-analysis	TNBC population, but intervention pooled across heterogeneous formulas	Systematic review/meta-analysis of 23 reported randomized trials	1,810 participants; 905 formula + chemotherapy vs. 905 chemotherapy; all reports from China	Heterogeneous Chinese medicinal formulas plus heterogeneous chemotherapy regimens vs. chemotherapy alone	ORR RR 1.39 (95% CI 1.28–1.52); recurrence RR 0.33; metastasis RR 0.48; QoL/KPS RR 1.55. PFS SMD 2.78 with I2 = 97%	Pooled reductions reported for gastrointestinal reactions (RR 0.59), myelosuppression (RR 0.61), and thrombocytopenia (RR 0.56); preparation-level AE attribution was not possible	Only 12/23 described random-number tables; allocation concealment and participant/personnel/outcome-assessor blinding unclear in all; formula heterogeneity; variable endpoints; possible publication bias	Not product-specific. Pooling does not establish efficacy or safety of any individual formula or commercial product

TNBC, relevance indicates how directly the population or model represents TNBC; it is not an evidence-strength score. Methodological robustness is judged separately within each design. Exact adverse-event counts are reported when available; “not reported” is not interpreted as absence of harm.

Abbreviations. AE, adverse event; CI, confidence interval; DFS, disease-free survival; HILI, herb-induced liver injury; HR, hazard ratio; IDFS, invasive disease-free survival; ORR, objective response rate; OS, overall survival; PFS, progression-free survival; QoL, quality of life; RR, risk ratio; RUCAM, roussel uclaf causality assessment method; SMD, standardized mean difference; TCM, traditional Chinese medicine; TNBC, triple-negative breast cancer.

## 3 Evidence synthesis

### 3.1 Potential antitumor mechanisms

Mechanistic findings are organized by biological process, but every claim is labeled by intervention class, TNBC relevance, and study design. TNBC-specific cell or animal evidence indicates disease relevance, not clinical efficacy. Reduced abundance or phosphorylation of a signaling protein is described as a pathway-associated effect unless binding, target-engagement, genetic rescue, or selective pharmacological validation establishes a direct molecular target.

#### 3.1.1 Regulation of cell death and proliferation

Programmed cell death and uncontrolled proliferation are central biological processes in TNBC progression. Selected Chinese medicine-derived metabolites and formulations may influence these processes, but the strength and directness of evidence vary across experimental contexts. Therefore, TNBC-specific and breast cancer-related studies should be prioritized when discussing direct antitumor effects, whereas findings from other tumor types or non-tumor models should be interpreted as extrapolated evidence.

Matrine is an isolated alkaloid present among the major constituents of Compound Kushen injection, but evidence for matrine alone cannot be attributed to the complete injection. In MDA-MB-231 and MDA-MB-468 cells, matrine reduced proliferation and invasion, increased apoptosis-associated readouts, and decreased PI3K/AKT pathway activity (Wei et al., 2023). These results support a pathway-associated relationship rather than direct PI3K or AKT inhibition because target engagement was not established. Sophoridine derivatives also induced apoptosis- and autophagy-associated changes with reduced mTOR-pathway signaling in TNBC models (Dai et al., 2022). Whether autophagy was cytotoxic or cytoprotective remains uncertain without flux analysis and functional rescue. These isolated-compound and derivative studies are hypothesis-generating and do not demonstrate the effects of a complete commercial product.

Compound Kushen injection is a complete commercial injectable preparation derived from Radix Sophorae Flavescentis (Kushen) and Rhizoma Heterosmilacis (Baituling) sources and contains multiple alkaloids, including matrine and oxymatrine. Breast cancer experiments and computational analyses have associated the complete preparation with cell-cycle, apoptosis, immune, metabolic, and tumor-microenvironment pathways (Cui et al., 2020; Li et al., 2021; Cui et al., 2019). Because several conclusions derive from computational prediction or incompletely stratified breast cancer models, they are classified as breast cancer-related and hypothesis-generating. Results for isolated matrine or oxymatrine are reported separately and are not used as surrogate proof for Compound Kushen injection.

#### 3.1.2 Ferroptosis

Ferroptosis is an iron-dependent form of regulated cell death characterized by membrane lipid peroxidation and is mechanistically distinct from apoptosis. Evidence in this review is centered on the complete commercial Shuganning injection rather than on an isolated constituent. In TNBC cells, Shuganning injection increased labile Fe2+, reactive oxygen species, and lipid-peroxidation readouts and produced cell death that was attenuated by ferrostatin-1; HO-1 dependence was also tested (Du et al., 2021). Tumor-growth suppression was reported in a nude-mouse xenograft, providing *in vivo* support but in an immune-deficient host.

This rescue evidence supports ferroptosis as a contributing death mechanism, but it does not establish direct binding to or selective inhibition of a molecular target. The studied product is licensed for hepatic indications rather than TNBC, and no TNBC clinical study has evaluated ferroptosis biomarkers, exposure-response relationships, or patient benefit for Shuganning injection. Ferroptosis is therefore presented as a preparation-specific preclinical mechanism, not a class effect or clinical claim.

#### 3.1.3 Modulation of TNBC-related oncogenic signaling pathways

TNBC progression involves PI3K/AKT/mTOR, MAPK, JAK/STAT3, NF-κB, and Wnt/β-catenin networks. The reviewed studies most often measured pathway-associated changes downstream of a complex preparation or isolated compound. Such changes may be biologically informative, but they do not by themselves establish direct target inhibition or explain which constituent, exposure, or cell state produced the effect.

In TNBC models, isolated matrine was associated with lower PI3K/AKT activity (Wei et al., 2023), sophoridine derivatives with lower mTOR-pathway activity (Dai et al., 2022), and Sanyin Formula plus paclitaxel with altered JAK/STAT3 signaling in a metastatic mouse model (Wang et al., 2023). In broader breast cancer models, Compound Kushen injection and isolated oxymatrine were associated with apoptosis, cell-cycle, tumor-microenvironment, and FAK/PI3K/AKT-related EMT changes (Cui et al., 2020; Li et al., 2021; Cui et al., 2019; Liu et al., 2022; Chen et al., 2019). These studies support pathway association, not direct inhibition of AKT, mTOR, FAK, or STAT3, unless the individual experiment included target engagement or a causal rescue.

Accordingly, decreases in phosphorylated AKT, mTOR, p70S6K, STAT3, or related mediators are described here as pathway-associated effects. Stronger causal language is reserved for experiments using binding assays, genetic knockdown or re-expression, pathway-specific rescue, or selective pharmacological controls. Claims involving p53/p21-mediated arrest also require model-specific qualification because TP53 alterations are common in TNBC. Evidence from unrelated disease systems is classified as extrapolated and is not used as primary support for anti-TNBC activity.

Therefore, evidence from unrelated disease models, such as icariin in osteoarthritis, Guanxinning tablet in cardiovascular disease, Huahong tablet in pelvic inflammatory disease, or EGFR/MAPK signaling in non-breast cancers, should not be used as primary support for anti-TNBC pathway regulation. Such studies may provide general mechanistic clues but should be classified as extrapolated evidence. Overall, current data suggest potential modulation of PI3K/AKT, JAK/STAT3, and Wnt/β-catenin-related networks, but further TNBC-specific validation using standardized CMBI preparations is required.

#### 3.1.4 Modulation of the tumor immune microenvironment

The tumor immune microenvironment plays a critical role in TNBC progression, therapeutic response, and immune escape. TNBC is generally more immunogenic than hormone receptor-positive breast cancer, but immune infiltration, checkpoint expression, macrophage polarization, and cytokine networks vary considerably among patients. Therefore, Chinese medicine-derived metabolites and formulations with immunomodulatory activity may have potential adjunctive value, particularly when their effects are evaluated in TNBC-specific or breast cancer-related models.

Immune-related evidence is heterogeneous in intervention class and experimental context. Cinobufagin is an isolated bufadienolide associated with, but not equivalent to, Huachansu/Cinobufacini products; in TNBC models it was associated with macrophage reprogramming and FAK/STAT3 pathway inactivation (Zhu et al., 2024). A four-botanical experimental formula plus doxorubicin was studied in the murine 4T1 syngeneic model, which preserves an immune-competent host but does not reproduce a human TNBC molecular subtype (Kan et al., 2024). Compound Kushen injection was examined in broader breast cancer models by single-cell RNA sequencing (Liu et al., 2022). These findings cannot be pooled as evidence for a single preparation class.

STAT3 activity can connect inflammatory signaling, macrophage state, cytokine production, and PD-L1 expression, but the reviewed preparation-level clinical studies did not test whether a CMBI changed tumor PD-1/PD-L1 biology or improved response to an immune checkpoint inhibitor. Thus, macrophage polarization, cytokine regulation, and checkpoint effects are presented as preclinical pathway-associated observations or hypotheses. A clinical immunotherapy claim would require a defined product, immune-relevant model validation, prespecified biomarkers, and prospective combination trials.

Evidence involving MARCO, PD-L1, CD47-targeting peptides, or inflammatory pathways in non-breast cancer models is considered extrapolated. Overall, selected preparations or isolated constituents may influence immune-associated readouts, but the evidence is primarily preclinical, compound-specific, and model-dependent. It does not establish a class effect of CMBIs or a clinical effect on immune-checkpoint therapy.

#### 3.1.5 Suppression of EMT, metastasis, and angiogenesis-related processes

Metastasis is a major cause of poor prognosis in TNBC and is closely associated with epithelial-mesenchymal transition (EMT), extracellular matrix remodeling, tumor-cell migration and invasion, angiogenesis, and interactions with the tumor microenvironment. Several Chinese medicine-derived metabolites and formulations have been investigated for their potential effects on these processes, but the directness of evidence varies substantially across studies.

TNBC-specific preclinical studies report several anti-metastatic associations, but the interventions are non-equivalent. Huaier polysaccharides are an isolated/enriched fraction and were reported to promote autophagic degradation of Snail (Tian et al., 2021). Sanyin Formula is a complete standardized trial formulation and was studied with paclitaxel in a mouse metastasis model (Wang et al., 2023). Cinobufagin is an isolated constituent and was associated with macrophage reprogramming and reduced FAK/STAT3 activity (Zhu et al., 2024). These findings support intervention-specific hypotheses and should not be generalized to commercial polyherbal products as a class.

EMT-related evidence commonly includes changes in E-cadherin, N-cadherin, vimentin, Snail, matrix metalloproteinases, or FAK-associated signaling. Huaier polysaccharide experiments included a mechanistically informative link to Snail degradation (Tian et al., 2021), whereas oxymatrine studies associated the isolated alkaloid with αVβ3-integrin/FAK/PI3K/AKT signaling in breast cancer cells (Chen et al., 2019). Direct target inhibition should not be inferred solely from altered expression or phosphorylation. Product-level confirmation, clinically plausible exposure, and rescue or target-engagement experiments remain necessary.

Breast cancer-related evidence also supports the relevance of EMT and invasion-related pathways. Oxymatrine was reported to reverse EMT in breast cancer cells by suppressing αVβ3 integrin/FAK/PI3K/AKT signaling (Chen et al., 2019). However, evidence for direct anti-angiogenic effects of CMBIs in TNBC remains limited. Findings from melanoma, colorectal cancer, ovarian cancer, or non-breast cancer angiogenesis models may provide mechanistic clues involving VEGF, MMPs, HIF-1α, or EMT-related pathways, but such evidence should be classified as extrapolated and should not be used as direct proof of anti-TNBC efficacy. Overall, current data suggest that selected Chinese medicine-derived interventions may affect EMT, invasion, metastasis, and angiogenesis-related signaling, but TNBC-specific validation using standardized CMBI preparations remains necessary.

#### 3.1.6 Regulation of metabolic reprogramming

Metabolic reprogramming is an important hallmark of TNBC, supporting rapid proliferation, invasion, survival under hypoxic stress, and therapeutic resistance. Altered glycolysis, mitochondrial function, lipid metabolism, amino acid metabolism, and redox homeostasis contribute to the aggressive phenotype of TNBC. Because many Chinese medicine formulations contain multiple bioactive metabolites, they may influence tumor metabolism through coordinated regulation of metabolic enzymes, stress-response pathways, and survival signaling. However, direct evidence in TNBC remains limited and should be distinguished from broader breast cancer-related or extrapolated evidence.

Compound Kushen injection was associated with suppressed cell-cycle, energy-metabolism, and DNA-repair programs in cancer cells, including MDA-MB-231 cells (Cui et al., 2019), but complete-product exposure should be distinguished from computationally inferred targets and from isolated Kushen alkaloids (Cui et al., 2020; Li et al., 2021). Cardamonin, an isolated chalcone rather than a commercial formula, was reported to reduce HIF-1α-dependent metabolic reprogramming in breast cancer models (Jin et al., 2019). These studies support intervention-specific metabolic hypotheses; they do not demonstrate a class effect or clinical metabolic modulation in TNBC.

#### 3.1.7 Enhancement of chemotherapy sensitivity

Chemotherapy remains an important systemic treatment backbone for many patients with TNBC, but intrinsic or acquired resistance frequently limits therapeutic efficacy. Potential mechanisms include enhanced DNA repair, altered cell-cycle regulation, apoptosis evasion, epithelial-mesenchymal transition, metabolic adaptation, cancer stem-like properties, and an immunosuppressive tumor microenvironment. Chinese medicine formulations may enhance chemotherapy sensitivity through multi-target regulation of these processes, but direct TNBC-specific evidence remains limited.

Drug-efflux transporters, including ABCB1, ABCG2, and ABCC1, can alter intracellular cytotoxic-drug exposure (Abd El-Aziz et al., 2021). However, transporter modulation by an isolated compound cannot be attributed to a complete CMBI. Moreover, an apparent tumor-cell sensitization effect could also change systemic pharmacokinetics or toxicity. Claims of resistance reversal therefore require a chemically defined intervention, transporter-specific functional assays, clinically plausible exposure, pharmacokinetic evaluation, and prospective safety monitoring.

Sanyin Formula plus paclitaxel produced antitumor and JAK/STAT3-associated effects in a TNBC metastasis mouse model (Wang et al., 2023). Compound Kushen injection was associated with chemotherapy response and tumor-environment changes in broader breast cancer models (Cui et al., 2019; Liu et al., 2022). These preclinical observations are compatible with chemotherapy sensitization but do not establish a clinical interaction. No reviewed TNBC trial prospectively demonstrated a pharmacokinetic or pharmacodynamic sensitization mechanism. Synthetic nanomedicine platforms were excluded as evidence for Chinese medicine-mediated resistance reversal.

#### 3.1.8 Critical appraisal: model diversity, mechanistic causality, and evidence quality

Frequent use of MDA-MB-231, MDA-MB-468, and 4T1 models creates a narrow representation of TNBC. MDA-MB-231 and MDA-MB-468 are human metastatic cell lines but represent different basal/mesenchymal states; 4T1 is a murine, highly metastatic mammary carcinoma used in immune-competent BALB/c mice (Lehmann et al., 2011; Dai et al., 2017; Pulaski and Ostrand-Rosenberg, 2001). A human xenograft in a nude mouse is immune-deficient, whereas a 4T1 syngeneic study retains murine immunity. These contexts cannot be treated as interchangeable evidence for macrophage, cytokine, or checkpoint mechanisms.

Model reporting should distinguish human from murine origin, immune-competent from immune-deficient hosts, primary from metastatic derivation, and basal-like/mesenchymal from other TNBC phenotypes. TP53, PTEN, BRCA1/2, androgen-receptor, and immune-context differences should be reported where relevant. Most studies reviewed here used two-dimensional culture or conventional xenografts; organoid and patient-derived model validation was largely absent. Therefore, disease relevance is highest only in a limited sense and does not confer high evidentiary strength.

Mechanistic confidence also varies. Ferrostatin-1 rescue in the Shuganning injection study and intervention-specific rescue approaches such as CXCL1 manipulation in XIAOPI research strengthen pathway-level inference (Du et al., 2021; Wang et al., 2019), but reduced phosphorylation alone does not prove direct target inhibition. Publication bias, incomplete product characterization, uncertain *in vivo* exposure, and limited independent replication may overstate consistency. Future studies should use model panels spanning TNBC phenotypes, include organoid or patient-derived validation, report negative findings, and combine dose-response, pharmacokinetic, target-engagement, genetic rescue, selective pharmacology, and independent replication.

### 3.2 Clinical evidence and application patterns

#### 3.2.1 Intervention classes and clinical application patterns

Clinical and translational evidence is grouped by intervention identity rather than under a single commercial-product category: (1) licensed/commercial multi-component products, (2) standardized trial formulations, (3) experimental multi-botanical mixtures, and (4) isolated constituents/fractions/derivatives. Only the first two classes have direct clinical relevance, and regulatory status may be for an indication other than TNBC. Product indication, manufacturer, dose, duration, and chemical characterization are reported when available.

Direct TNBC clinical evidence includes a multicenter randomized trial of the standardized Sanyin Formula plus chemotherapy (Wu et al., 2022), a single-center randomized trial of XIAOPI Formula after surgery and chemotherapy (Guo et al., 2024), and a prospective observational cohort of individualized Chinese medicine formulas (Wang et al., 2020). These studies evaluated different products, populations, and endpoints and should not be interpreted as replicates of one intervention. Their relevance to TNBC is high, but methodological robustness ranges from randomized blinded designs to an open observational exposure.

Compound Kushen injection, Kanglaite injection, Huachansu/Cinobufacini products, and Pingxiao capsules have broader oncology or breast-cancer literature but limited strictly defined TNBC clinical evidence. Their use, regulation, composition, and manufacturing are product-specific. Results from isolated constituents such as matrine or cinobufagin are not used as clinical evidence for these complete products.

#### 3.2.2 Clinical endpoint and evidence limitations

TNBC clinical studies remain few and clinically heterogeneous. Sanyin Formula primarily evaluated disease-free survival, XIAOPI Formula emphasized CXCL1 and patient-reported outcomes with exploratory long-term disease-free survival, and the Wang cohort evaluated recurrence/metastasis under non-randomized exposure (Wu et al., 2022; Guo et al., 2024; Wang et al., 2020). These endpoints, treatment settings, and designs are not directly comparable.

A 2022 meta-analysis of 23 reported randomized trials (1,810 participants) comparing Chinese medicinal formulas plus chemotherapy with chemotherapy alone reported favorable pooled estimates for objective response (RR 1.39, 95% CI 1.28–1.52), recurrence (RR 0.33), metastasis (RR 0.48), performance status, and several toxicity outcomes (Zhang et al., 2022). However, all included reports were from China; formulas and chemotherapy regimens were heterogeneous; only 12 trials explicitly described a random-number table; allocation concealment and blinding were unclear in all trials; and progression-free survival showed extreme heterogeneity (I^2^ = 97%). The meta-analysis therefore broadens the clinical context but does not overcome the insufficient quality of the primary trials or establish efficacy for any specific product.

#### 3.2.3 Representative studies

The available TNBC studies provide preliminary, intervention-specific signals rather than a coherent clinical evidence base. Sanyin Formula plus chemotherapy improved 5-year disease-free survival in one randomized trial without a significant overall-survival difference (Wu et al., 2022). XIAOPI Formula changed CXCL1 and patient-reported outcomes in a small randomized trial, with exploratory long-term disease-free survival findings (Guo et al., 2024). The observational formula cohort reported an association with lower recurrence/metastasis risk, but confounding, attrition, individualized formula modification, and non-randomized exposure limit causal inference (Wang et al., 2020). Table 2 separates TNBC relevance from study design and methodological concerns.

Evidence for other commercial oncology products should not be presented as representative TNBC clinical evidence unless the enrolled TNBC population, exact marketed product, manufacturer/batch, dose, comparator, concomitant therapy, endpoints, and adverse events are documented. Future studies should also report molecular subtype or biomarker context and distinguish recurrence-prevention, metastatic, and symptom-management settings.

### 3.3 Current challenges and translational bottlenecks

#### 3.3.1 Limited high-quality clinical evidence

The direct TNBC evidence comprises two small randomized trials with different primary outcomes and one observational cohort (Wu et al., 2022; Guo et al., 2024; Wang et al., 2020). The broader meta-analysis of 23 reported randomized trials suggests possible benefits but is constrained by unclear concealment/blinding, heterogeneous formulas and chemotherapy regimens, variable endpoints, and high heterogeneity for several outcomes (Zhang et al., 2022). Relevance to TNBC therefore does not equate to low risk of bias or high certainty. The current evidence is insufficient to support a class-wide efficacy conclusion.

Future trials should be multicenter and prospectively registered, with concealed randomization, credible blinding where feasible, prespecified clinically meaningful endpoints, adequate power, complete follow-up, and standardized adverse-event reporting. Each study should identify the exact product or formula, manufacturer, batch or fingerprint, composition, dose, duration, and concomitant anticancer therapy (Schulz et al., 2010; Gagnier et al., 2006).

#### 3.3.2 Product identity, standardization, and quality control

Product-level information is essential because the reviewed interventions differ in composition and regulatory status. Compound Kushen injection and Shuganning injection are complete commercial injectable products, whereas Sanyin Formula is a standardized trial formula, XIAOPI Formula has a separate non-oncology regulatory history, the four-botanical formula is experimental, and matrine, oxymatrine, sophoridine derivatives, cinobufagin, cardamonin, and Huaier polysaccharides are isolated constituents, derivatives, or fractions. These categories are displayed separately in Table 1 and are not treated as interchangeable evidence.

For the principal complete preparations, reports should provide botanical sources, extraction and manufacturing process, manufacturer, batch number, dose, administration schedule, approved indication, and chromatographic fingerprint or multi-marker quantification. Sanyin Formula was administered as manufacturer-supplied granules at 14 g twice daily for at least 2 years in the pivotal trial (Wu et al., 2022). XIAOPI Formula is a ten-material reflux extract with reported HPLC fingerprinting and was approved in China for mammary hyperplasia rather than TNBC (Wang et al., 2019). Compound Kushen injection and Shuganning injection have commercial product identities, but the oncology studies reviewed do not consistently report batch-level chemical comparability. Quality control must therefore accompany exposure, pharmacokinetic, pharmacodynamic, and safety evaluation; *in vitro* concentrations should not be assumed clinically achievable (Liang et al., 2009; Liu et al., 2025; Alsanad et al., 2014).

#### 3.3.3 Lack of predictive and pharmacodynamic biomarkers

Validated predictive and pharmacodynamic biomarkers are lacking for all intervention classes. Conventional endpoints do not identify which patients might benefit or whether a proposed pathway was engaged at a clinically achievable exposure. Biomarker interpretation must also account for TNBC molecular heterogeneity rather than assuming that a result from one cell line represents the entire disease.

The XIAOPI program linked the intervention to CXCL1-associated readouts and used experimental manipulation to support pathway inference (Wang et al., 2019; Guo et al., 2024). Future studies could prespecify PD-L1, tumor-infiltrating lymphocytes, BRCA1/2, circulating tumor DNA, inflammatory cytokines, metabolomic profiles, or immune-cell signatures (Bianchini et al., 2022; Davis et al., 2025). These biomarkers should be tied to a chemically defined product, relevant model, and clinical endpoint rather than used as exploratory confirmation after the fact.

#### 3.3.4 Inadequate safety monitoring and botanical drug-drug interaction assessment

Preparation-specific safety reporting is sparse but not absent. In the Sanyin trial, treatment-related adverse events occurred in 3/127 participants receiving Sanyin Formula and 4/125 receiving placebo; events included upper-limb edema, pain, and diarrhea in the Sanyin group, with no treatment-related deaths or life-threatening events (Wu et al., 2022). In the XIAOPI trial, specific adverse-event counts, categories, and grades were not reported; the authors stated only that the formula had few side effects (Guo et al., 2024). In the observational formula cohort, 6/73 exposed patients experienced reported adverse events, including one severe herb-induced liver injury (RUCAM score 6), two mild transaminase elevations, two cases of stomach pain, and one case of diarrhea (Wang et al., 2020). These data do not establish comparative safety across preparations because products, designs, denominators, and ascertainment differed.

Future trials should prospectively record adverse events with standardized criteria, causality assessment, laboratory monitoring, dose interruption, discontinuation, and long-term follow-up. Preparation-specific interaction studies are needed for chemotherapy, immune checkpoint inhibitors, PARP inhibitors, antibody-drug conjugates, and supportive medicines. General herb-drug interaction reviews (Alsanad et al., 2014) provide context but cannot substitute for product-specific pharmacokinetic and safety data. Additional safety and botanical drug-drug interaction considerations are summarized in Supplementary Table 2.

### 3.4 Limitations of this review

This narrative review does not provide an exhaustive systematic search, formal risk-of-bias tool, or certainty grade. The two-dimensional framework separates TNBC relevance from study design and uses design-appropriate methodological domains, but the judgments remain narrative and may be affected by study selection and reporting limitations.

Interventions were highly heterogeneous and included commercial products, standardized trial formulas, experimental mixtures, isolated compounds, derivatives, and fractions. The umbrella term CMBI is used only for mapping; no class-wide pharmacological or regulatory equivalence is assumed. Product composition, dose, and quality-control information were incomplete in several reports.

Clinical evidence is limited to a few TNBC-specific studies with dissimilar endpoints. The larger meta-analysis (Zhang et al., 2022) broadens the evidence base but contains heterogeneous formulas and chemotherapy regimens and predominantly unclear risk-of-bias domains. Conclusions about survival, chemotherapy sensitization, quality of life, or toxicity reduction therefore remain preliminary and product-specific.

Preclinical evidence is concentrated in MDA-MB-231, MDA-MB-468, and 4T1 models and rarely includes organoid or patient-derived validation. Publication bias and the predominance of positive pathway readouts may overstate mechanistic consistency. Direct target claims were therefore restricted to studies with causal validation, and most findings are described as pathway-associated.

## 4 Conclusion

Chinese medicine-based interventions comprise non-equivalent commercial products, standardized trial formulations, experimental mixtures, isolated constituents, derivatives, and fractions. Selected interventions show pathway-associated effects on apoptosis, ferroptosis, EMT, immune-related readouts, metabolism, or chemotherapy response, but most evidence is preclinical and model-dependent. Ferroptosis is supported primarily by one Shuganning injection program with iron/lipid-peroxidation and ferrostatin-1 rescue evidence (Du et al., 2021); immune-checkpoint and chemotherapy-sensitization claims remain indirect or unvalidated clinically. A small number of TNBC clinical studies and a methodologically limited meta-analysis provide preliminary adjunctive signals without establishing a class effect (Wu et al., 2022; Guo et al., 2024; Wang et al., 2020; Zhang et al., 2022).

No CMBI should currently be regarded as an established TNBC-specific therapy. Future translation requires exact product identity, batch-level chemical characterization, clinically relevant exposure, model panels spanning TNBC phenotypes and immune contexts, organoid or patient-derived validation, causal target experiments, multicenter randomized trials, and preparation-specific adverse-event and drug-interaction monitoring. Until such evidence is available, conclusions should remain intervention-specific, adjunctive, and explicitly qualified by study design and methodological limitations.
