# Supplementary material for: Chinese medicine-based interventions in triple-negative breast cancer: mechanistic evidence, clinical findings, and translational challenges-a narrative review
Source: Front Pharmacol. 2026 Sep 9;17:1787613. doi: 10.3389/fphar.2026.1787613 (PMC13598637; doi:10.3389/fphar.2026.1787613)
Supplement: Supplementary file 1 [file Table1.docx]

**Supplementary Table 1. Composition principal Chinese medicine-based interventions discussed in the review incl. taxonomically valid species**

| **Item discussed in the review** | **Category** | **Botanical, fungal, or animal source material** | **Plant or natural metabolite / preparation** | **Validated taxonomic name with authority** | **Family** | **Taxonomic source used** | **Notes on evidence relevance and limitations** |
| --- | --- | --- | --- | --- | --- | --- | --- |
| Compound Kushen injection | Commercial Chinese polyherbal preparation (CCPP); injectable preparation | Kushen | Alkaloid-rich preparation; matrine and oxymatrine are major metabolites | *Sophora flavescens Aiton* | Fabaceae | MPNS; POWO | Reported botanical source for Kushen. Evidence in the review is mainly breast cancer-related or systems-pharmacology evidence, with limited direct TNBC clinical validation. |
| Compound Kushen injection | CCPP; injectable preparation | Baituling | Botanical drug material in Compound Kushen injection | *Heterosmilax chinensis F.T.Wang* | Smilacaceae | MPNS; POWO | Reported source material for Baituling in Compound Kushen injection. Product- and batch-specific documentation should be provided in future studies. |
| Matrine | Plant metabolite; Kushen-derived alkaloid | Kushen | Matrine | *Sophora flavescens Aiton* | Fabaceae | MPNS; POWO | Used as TNBC-specific plant metabolite-level evidence. It should not be equated directly with complete-product effects without formulation-level validation. |
| Oxymatrine | Plant metabolite; Kushen-derived alkaloid | Kushen | Oxymatrine | *Sophora flavescens Aiton* | Fabaceae | MPNS; POWO | Used as breast cancer-related mechanistic evidence for EMT and integrin/FAK/PI3K/AKT-associated regulation. |
| Sophoridine derivatives | Plant metabolite derivatives | Sophora source | Sophoridine and derivatives | *Sophora alopecuroides L.* | Fabaceae | MPNS; POWO | Derivative-level TNBC preclinical evidence. These data support mechanistic plausibility but do not represent standardized complete-product-level evidence. |
| Shuganning injection | CCPP; injectable preparation | Yinchen / Artemisia source | Multi-constituent preparation | *Artemisia capillaris Thunb.* | Asteraceae | MPNS; POWO | One reported botanical source of Shuganning injection. TNBC evidence relates to ferroptosis induction, but formulation standardization and clinical validation remain necessary. |
| Shuganning injection | CCPP; injectable preparation | Zhizi / Gardenia source | Multi-constituent preparation; iridoid-related metabolites may be present | *Gardenia jasminoides J.Ellis* | Rubiaceae | MPNS; POWO | Reported botanical source of Shuganning injection. Product-specific composition and batch consistency should be reported. |
| Shuganning injection | CCPP; injectable preparation | Huangqin / Scutellaria source | Multi-constituent preparation; baicalin-related metabolites may be present | *Scutellaria baicalensis Georgi* | Lamiaceae | MPNS; POWO | Reported botanical source of Shuganning injection. Mechanistic findings should be interpreted in relation to standardized preparation data. |
| Huaier polysaccharides | Fungal-derived preparation | Huaier | Polysaccharide fraction | *Trametes robiniophila Murrill* | Polyporaceae | MycoBank; Index Fungorum | Non-botanical fungal source included for transparency. Used as TNBC-specific preclinical evidence for Snail-related EMT suppression. |
| Cardamonin | Plant metabolite | Alpinia source | Cardamonin | *Alpinia katsumadai Hayata* | Zingiberaceae | MPNS; POWO | Used as broader breast cancer-related natural metabolite evidence for HIF-1alpha-dependent metabolic reprogramming; not direct complete-product or TNBC clinical evidence. |
| Kanglaite injection | CCPP; injectable preparation | Yiren / Coix seed source | Coix seed oil-related preparation | *Coix lacryma-jobi var. ma-yuen (Rom.Caill.) Stapf* | Poaceae | MPNS; POWO | Mentioned as a clinically used oncology CCPP. The review does not treat it as direct TNBC-specific clinical evidence. |
| Huachansu / Cinobufacini preparations | Animal-derived commercial preparation | Chan Su / toad venom-related source | Bufadienolide-containing preparation | *Bufo gargarizans Cantor* | Bufonidae | NCBI Taxonomy; Amphibian Species of the World | Non-botanical source. Included because cinobufagin-related evidence is discussed; product identity and safety monitoring are essential. |
| Cinobufagin | Animal-derived natural metabolite | Chan Su / toad venom-related source | Cinobufagin | *Bufo gargarizans Cantor* | Bufonidae | NCBI Taxonomy; Amphibian Species of the World | Used as TNBC-specific preclinical evidence for macrophage reprogramming and FAK/STAT3-related metastasis regulation; not botanical. |
| Sanyin Formula | Chinese medicine formula | Multi-botanical formula | Formula-level preparation | Not taxonomically expanded in the revised manuscript | Not applicable | Primary clinical and preclinical publications; product documentation required | Used as TNBC-specific clinical and preclinical formula evidence. Future reports should provide the complete species list, plant parts, authorities, families, voucher information, and batch records. |
| XIAOPI Formula | Chinese medicine formula | Multi-botanical formula | Formula-level preparation | Not taxonomically expanded in the revised manuscript | Not applicable | Primary clinical publication; product documentation required | Used as TNBC-specific clinical evidence. Future studies should report full botanical identities, authorities, families, dosage, and quality control information. |
| Four-herb formula combined with doxorubicin | Chinese medicine formula | Multi-botanical formula | Formula-level preparation | Not taxonomically expanded in the revised manuscript | Not applicable | Primary preclinical publication; product documentation required | Used as breast cancer-related preclinical evidence. Full taxonomic details should be provided in studies using the formula. |
| Pingxiao capsules | Commercial multi-source preparation | Commercial product mentioned as broader oncology example | Multi-source preparation | Not taxonomically expanded in the revised manuscript | Not applicable | Product-specific pharmacopeial or manufacturer documentation required | Mentioned only as a broader oncology commercial product, not as direct TNBC-specific evidence. Complete botanical, animal, and mineral source documentation is needed if evaluated in future TNBC studies. |

This table improves ethnopharmacological transparency. Botanical names were checked against MPNS and POWO; fungal and animal-derived materials were checked against MycoBank/Index Fungorum, NCBI Taxonomy, or Amphibian Species of the World, as applicable. For proprietary or multi-botanical formulas whose complete composition was not available, product-specific documentation is requested rather than inferred. Database access date: August 7, 2026. MPNS, https://mpns.science.kew.org/mpns-portal/; POWO, https://powo.science.kew.org/; MycoBank, https://www.mycobank.org/; Index Fungorum, https://www.indexfungorum.org/; NCBI Taxonomy, https://www.ncbi.nlm.nih.gov/taxonomy; Amphibian Species of the World, https://amphibiansoftheworld.amnh.org/.

Abbreviations. CMBI, Chinese medicine-based intervention; EMT, epithelial–mesenchymal transition; FAK, focal adhesion kinase; HIF-1α, hypoxia-inducible factor 1α; MPNS, Medicinal Plant Names Services; POWO, Plants of the World Online; TNBC, triple-negative breast cancer.
