# Supplementary material for: Chinese medicine-based interventions in triple-negative breast cancer: mechanistic evidence, clinical findings, and translational challenges-a narrative review
Source: Front Pharmacol. 2026 Sep 9;17:1787613. doi: 10.3389/fphar.2026.1787613 (PMC13598637; doi:10.3389/fphar.2026.1787613)
Supplement: Supplementary file 2 [file Table2.docx]

**Supplementary Table 2. Safety and botanical drug–drug interaction considerations for Chinese medicine-based interventions in TNBC**

| **Safety domain** | **Potential concern** | **Relevance to TNBC treatment** | **Current evidence limitation** | **Recommended reporting in future studies** |
| --- | --- | --- | --- | --- |
| Liver and renal function | Hepatotoxicity, nephrotoxicity, abnormal liver enzymes, renal impairment, or altered clearance of concomitant anticancer drugs. | TNBC patients may receive chemotherapy, immune checkpoint inhibitors, PARP inhibitors, antibody-drug conjugates, analgesics, antiemetics, or antibiotics, many of which require hepatic or renal monitoring. | Most available CMBI-related TNBC studies provide limited longitudinal laboratory monitoring and do not clearly separate CMBI-related toxicity from conventional treatment toxicity. | Report baseline and serial ALT, AST, bilirubin, creatinine, estimated glomerular filtration rate, dose modification, treatment interruption, and attribution of adverse events. |
| Hematologic toxicity | Myelosuppression, anemia, neutropenia, thrombocytopenia, or overlapping marrow toxicity with cytotoxic therapy. | Chemotherapy regimens used in TNBC commonly cause hematologic toxicity; adjunctive formulations may either modify tolerance or complicate toxicity attribution. | Clinical studies rarely provide detailed grade-specific hematologic adverse events or predefined management rules. | Report complete blood counts, CTCAE grade, timing relative to chemotherapy cycles, supportive care, dose delays, and discontinuation. |
| Gastrointestinal reactions | Nausea, vomiting, diarrhea, constipation, mucositis, anorexia, abdominal discomfort, or dehydration. | Gastrointestinal toxicity affects treatment adherence and quality of life in patients receiving chemotherapy or supportive medications. | Current reports often focus on efficacy endpoints and incompletely describe gastrointestinal adverse events and severity. | Record symptom frequency, severity, duration, CTCAE grade, rescue medication use, hydration status, and effect on treatment adherence. |
| Allergic and infusion-related reactions | Rash, pruritus, urticaria, fever, hypersensitivity, anaphylaxis, or injection-related reactions. | Injectable CMBIs or animal-derived preparations may carry risk of hypersensitivity, especially when combined with taxanes, platinum agents, or immune therapies. | Premedication, infusion protocols, causality assessment, and recurrence on rechallenge are often insufficiently documented. | Report route of administration, infusion rate, premedication, timing of reaction, severity, management, rechallenge, and causality assessment. |
| Cardiovascular events | Palpitations, blood pressure changes, arrhythmia, thromboembolic events, cardiac dysfunction, or vascular events. | TNBC treatment may include anthracyclines and other agents with cardiovascular risk; adjunctive preparations may add uncertainty if monitoring is incomplete. | Cardiovascular outcomes are rarely prespecified in CMBI-related TNBC studies. | Report cardiovascular history, electrocardiogram when relevant, blood pressure, cardiac biomarkers or echocardiography when indicated, and serious adverse events. |
| Immune-related adverse events | Pneumonitis, colitis, hepatitis, endocrinopathy, dermatitis, or immune activation when used with immune checkpoint inhibitors. | Immunotherapy is increasingly used in TNBC; immunomodulatory CMBIs may theoretically influence immune activity or toxicity profiles. | Direct clinical evidence for CMBIs combined with immune checkpoint inhibitors in TNBC is limited. | Document immunotherapy exposure, immune-related adverse events, steroid or immunosuppressive treatment, treatment interruption, and immune biomarker status when available. |
| CYP enzyme interactions | Inhibition or induction of CYP enzymes may alter exposure to paclitaxel, cyclophosphamide, anthracyclines, PARP inhibitors, antiemetics, anticoagulants, or supportive drugs. | Patients with TNBC frequently receive multiple drugs; metabolic interactions may affect efficacy or toxicity. | Most clinical studies do not include enzyme assays, pharmacokinetic sampling, or systematic concomitant medication review. | Report concomitant medications, suspected CYP substrates, inhibitors or inducers, pharmacokinetic parameters, dose changes, toxicity signals, and interaction assessment. |
| ABC transporter interactions | Modulation of P-gp/ABCB1, ABCG2/BCRP, ABCC1/MRP1, or related transporters may alter intracellular or systemic drug exposure. | Transporter modulation may appear to enhance chemosensitivity in vitro but may also change tissue distribution and toxicity in patients. | Evidence in TNBC remains mainly mechanistic or extrapolated; transporter effects are rarely linked to clinical pharmacokinetics. | Report transporter assays when relevant, drug exposure data, tumor and plasma concentrations if available, toxicity monitoring, and clinical endpoints. |
| Pharmacokinetics and pharmacodynamics | Changes in absorption, distribution, metabolism, excretion, bioavailability, active metabolites, target engagement, or pharmacodynamic response. | Complex formulations may have variable composition and exposure, making dose-effect and exposure-effect relationships difficult to interpret. | Many studies do not report dose rationale, bioavailability, active metabolites, tissue distribution, or pharmacodynamic biomarkers. | Report formulation dose, schedule, route, active marker substances, PK parameters, PD biomarkers, exposure-response analyses, and safety margins. |
| Combination with anticancer therapy | Overlapping toxicity or altered efficacy with chemotherapy, immunotherapy, PARP inhibitors, antibody-drug conjugates, endocrine or supportive medications. | TNBC treatment is increasingly multimodal; uncontrolled concomitant therapy can confound efficacy and safety interpretation. | Current clinical evidence is limited by heterogeneous background therapy and incomplete reporting of concomitant medications. | Specify treatment stage, anticancer regimen, line of therapy, dose intensity, concomitant drugs, supportive care, adverse events, and predefined stopping rules. |
| Formulation quality and reproducibility | Batch variability, uncertain botanical or animal source identity, inconsistent marker substances, contamination, adulteration, or variable dosing. | Safety signals may differ across products, batches, routes of administration, and manufacturing standards. | Product composition, batch number, quality control, and taxonomic validation are often incompletely reported. | Report product name, manufacturer, batch number, source authentication, quality control tests, marker substances, contaminants, storage, and preparation process. |

This table provides a prospective framework for safety and botanical drug–drug interaction reporting. It complements, but does not replace, the preparation-specific adverse-event data reported in Manuscript Table 2. It does not imply confirmed safety risks for any product.

Abbreviations. ABCB1, ATP-binding cassette subfamily B member 1; ABCG2, ATP-binding cassette subfamily G member 2; ADC, antibody–drug conjugate; ALT, alanine aminotransferase; AST, aspartate aminotransferase; BCRP, breast cancer resistance protein; CMBI, Chinese medicine-based intervention; CTCAE, Common Terminology Criteria for Adverse Events; CYP, cytochrome P450; PARP, poly(ADP-ribose) polymerase; PD, pharmacodynamic; PK, pharmacokinetic; TNBC, triple-negative breast cancer.
